# Tissue-Engineered Skeletal Muscle Models to Study Muscle Function, Plasticity, and Disease

**DOI:** 10.3389/fphys.2021.619710

**Published:** 2021-02-26

**Authors:** Alastair Khodabukus

**Affiliations:** Department of Biomedical Engineering, Duke University, Durham, NC, United States

**Keywords:** skeletal muscle, tissue engineering, fiber-type, satellite cell, disease modeling, Duchenne Muscle dystrophy, innervation, myosin heavy chain

## Abstract

Skeletal muscle possesses remarkable plasticity that permits functional adaptations to a wide range of signals such as motor input, exercise, and disease. Small animal models have been pivotal in elucidating the molecular mechanisms regulating skeletal muscle adaptation and plasticity. However, these small animal models fail to accurately model human muscle disease resulting in poor clinical success of therapies. Here, we review the potential of *in vitro* three-dimensional tissue-engineered skeletal muscle models to study muscle function, plasticity, and disease. First, we discuss the generation and function of *in vitro* skeletal muscle models. We then discuss the genetic, neural, and hormonal factors regulating skeletal muscle fiber-type *in vivo* and the ability of current *in vitro* models to study muscle fiber-type regulation. We also evaluate the potential of these systems to be utilized in a patient-specific manner to accurately model and gain novel insights into diseases such as Duchenne muscular dystrophy (DMD) and volumetric muscle loss. We conclude with a discussion on future developments required for tissue-engineered skeletal muscle models to become more mature, biomimetic, and widely utilized for studying muscle physiology, disease, and clinical use.

## Introduction

Skeletal muscle is the largest organ in the body by mass and is essential for respiration, locomotion, posture, and whole-body energy homeostasis. To attain maximal performance and efficiency for these diverse roles, skeletal muscle displays a remarkable level of plasticity. Specifically, multiple isoforms of contractile, calcium-handling, metabolic, and structural proteins have evolved to meet the broad demands placed upon skeletal muscle (Schiaffino and Reggiani, 2011). Skeletal muscle dysfunction due to genetic mutations, aging, volumetric muscle loss, or acquired diseases significantly impair quality of life and can even be lethal. The foundation of our mechanistic understanding of skeletal muscle function, plasticity, and disease is derived predominantly from *in vivo* animal experiments and two-dimensional (2D) *in vitro* cell culture studies. Small animal studies, particularly comparative biology and genetic manipulations, have been pivotal in elucidating the molecular mechanisms regulating skeletal muscle function and plasticity (Schiaffino and Reggiani, 2011; Hoppeler, 2016). However, small animal models require additional translational and validation models to increase the successful translation of identified therapies to the clinic (Hay et al., 2014). The *in vitro* culture of human cells has the potential to generate experimental models with increased translational relevance. However, traditional skeletal muscle cell culture systems have limited longevity due to cellular detachment resulting in developmentally immature tissues with limited translational relevance. Over the past 30 years, three-dimensional (3D) tissue engineered skeletal muscle culture systems have been developed that better mimic the native muscle microenvironment, permit functional testing, and enable prolonged culture durations (Khodabukus et al., 2018; Wang J. et al., 2019). In this review, we discuss methods to generate three-dimensional tissues and factors that regulate their functionality. We then discuss multiple factors regulating skeletal muscle fiber-type *in vivo* and the ability to study these factors *in vitro*. Lastly, we discuss further developments regulated for engineered muscle tissues to become more widely utilized and to better model adult skeletal muscle.

## Skeletal Muscle Structure and Function

### Skeletal Muscle Structure

Skeletal muscle is comprised of a hierarchical architectural structure that permits efficient force generation. Ultra-structurally, the most basic units of a myofibers are sarcomeres that contain myosin and actin which form overlaps to permits muscle contraction in a calcium-dependent manner. Efficient muscle contraction is coordinated by the transverse tubule system, a branched membrane network that runs along the entire length of the myofiber to the junction of the A and I bands of the sarcomere. The calcium required for contraction is stored in the sarcoplasmic reticulum (SR) which connects to the t-tubule at the specialized terminal cisternae. The SR stores calcium at a significantly higher concentration than seen in the sarcoplasm due to the calcium-binding protein calsequestrin (CSQ) (Murphy et al., 2009; Lamboley et al., 2013). Calcium is released from SR by the ryanodine receptor 1 (RyR1) into the myofibrils and binds to troponin C inducing a conformational change which results in removal of tropomyosin from myosin which enables actin to bind to myosin. Actin binding to myosin results in adenosine triphosphate (ATP) hydrolysis that causes actin to pull along myosin, shortening the sarcomere and generating muscle contraction (Sellers, 2004). Skeletal muscle relaxation is an active process that requires removal of calcium from the myofibrils to reestablish tropomyosin blocking of myosin actin-binding sites. Calcium removal is regulated by the sarcoplasmic-endoplasmic reticulum Ca^2+^ ATPase pumps (SERCA) that pump calcium back to the SR and the high affinity calcium binding protein parvalbumin found in fast skeletal muscle that quickens relaxation rate (Muntener et al., 1985; Schwaller et al., 1999).

## Skeletal Muscle Culture Models

### Skeletal Muscle Explants and Single Fiber Cultures

Due to the high metabolic demands of skeletal muscle, long-term *in vitro* culture of whole skeletal muscles is impossible due to hypoxia and resultant loss of viability. To minimize hypoxic stress, muscles such as the extensor digitorum longus (EDL) and soleus (SOL) can be dissected to generate muscle strips that can be cultured for up to 12 h in highly oxygenated media (Park et al., 2012). These tissues can be utilized to measure contractile function (Brooks and Faulkner, 1988) and assess insulin-stimulated glucose uptake (Hansen et al., 1994). To overcome the short-term culture duration of intact muscle explants, single myofibers can be carefully dissected and isolated from the muscle belly and cultured for up to 7 days (Renzini et al., 2018), though most studies utilize 48–96 h time points. The single myofiber model is the gold-standard model to study satellite cell activation *in vitro*, due to SCs being retained within their niche and the ability to study SC dynamics with multiple modalities (Pasut et al., 2013; Wang Y. X. et al., 2019). Single fiber studies utilizing transgenic mice have been used to help unravel the transcription factors and molecular mechanisms regulating SC activation (Beauchamp et al., 2000; Kuang et al., 2007), polarity (Le Grand et al., 2009; Dumont et al., 2015), and symmetric divisions (Wang Y. X. et al., 2019). Intact single fiber studies have also enabled role of MyHC isoform on contractile properties (Bottinelli et al., 1996; Harridge et al., 1996) and the factors regulating muscle fatigue to be assessed (Westerblad and Allen, 1991; Westerblad et al., 1993). Single myofibers can also be used to assess multiple aspects of muscle physiology includingfactors regulating excitation-coupling (Allen et al., 1997; Prosser et al., 2010), genetic regulators of calcium-handling and t-tubule stability (Al-Qusairi et al., 2009; Kerr et al., 2013), membrane resealing in response to injury (Bansal et al., 2003; Sreetama et al., 2018), glucose uptake in response to electrical stimulation (Castorena et al., 2015) and insulin (Lanner et al., 2006), and to study mitochondrial function (Schuh et al., 2012). Single fiber studies have been pivotal to increasing our understanding of muscle physiology and permit *in vitro* testing of muscle fibers with adult ultrastructure and function. Importantly, the described 2D and 3D studies below are yet to replicate the developmental maturation and contractile function of the single fiber system. However, both explant and single fiber preparations are technically challenging, have limited experimental duration, are difficult to scale up for high throughput screening, and require continual new samples – making large-scale experiments in human tissues infeasible.

### Traditional Two-Dimensional Models

The alternative method to single fiber culture models is to liberate satellite cells from their niche by enzymatic dissociation or permitting them to “outgrow” from partially minced muscle tissue. This method results in heterogenous cell populations found within skeletal muscles that can be further purified by cell surface marker expression (Maesner et al., 2016; Uezumi et al., 2016) by taking advantage of the faster adhesion kinetics of fibroblasts to enrich for muscle progenitor cells by pre-plating (Gharaibeh et al., 2008). Satellite cells isolated by this method rapidly activate and become myoblasts or muscle precursor cells (MPCs) characterized by increased expression of MyoD, and decreased expression of Pax7 (Ryall et al., 2015). The functional impact of this SC activation is the dramatically impaired ability of these cultured cells to engraft in skeletal muscle upon transplantation, with just 72 h in culture decreasing engraftment efficiency threefold (Montarras et al., 2005). To minimize activation of these cells, small molecules (Charville et al., 2015), culture substratum stiffness (Gilbert et al., 2010), and regulating metabolic fuel source (Ryall et al., 2015) have been described to maintain MPCs in a more stem-like state. Alternatively, a quiescence media and culture system has been developed that can maintain SCs in a quiescent-like state but cannot reverse activated cells into a quiescent state (Quarta et al., 2016).

After expansion, MPCs can be induced to differentiate by reduction of serum content that induces cell cycle withdrawal, upregulation of differentiation genes, and ultimately fusion into multi-nucleated myotubes. Due to the rapid rate (48–96 h) of fusion and ease of the system, this model is the most frequently used system to assess the impact of genetic manipulations, growth factors, or small molecules on muscle differentiation and fusion. However, the assessment of longer-term (<7 days) experimental interventions are often prevented due to the detachment of myotubes due to spontaneous contractions of developing myotubes (Cooper et al., 2004). Furthermore, this detachment limits the developmental maturation of the tissue cultures, limiting the translation relevance of experimental findings (Rao et al., 2018). Lastly, the assessment of contractile function, the primary measure for therapeutic efficacy, is not permitted by traditional two-dimensional culture preventing functional assessments.

### Engineered Skeletal Muscle Models

**FIGURE 1:**
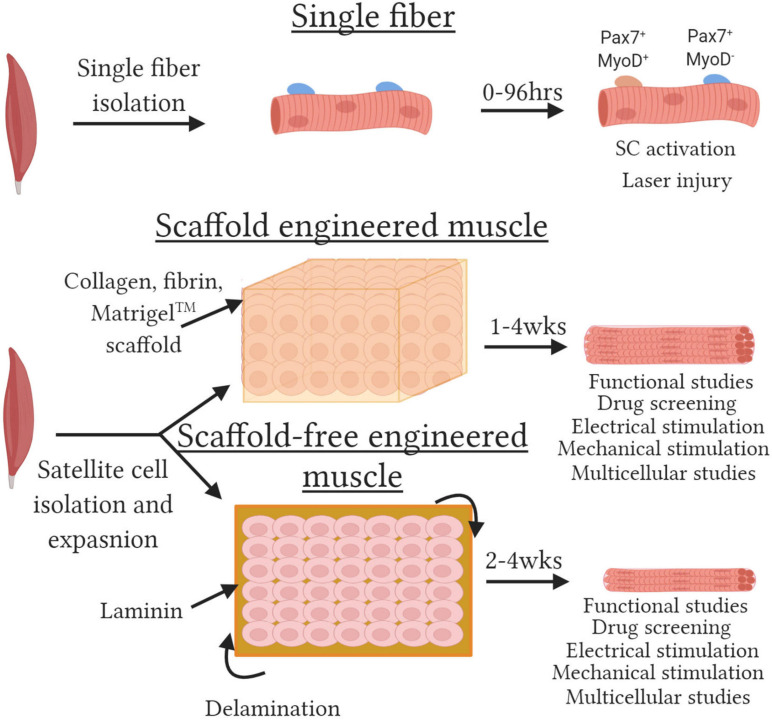
Methods to engineered functional skeletal muscle. Schematic depicting various skeletal muscle models. **(Top)** Single fibers with satellite cells retained within their niche are isolated by enzymatic and mechanical dissociation. Single fibers can be cultured for up to 120 h and satellite cell activation assessed by evaluating Pax7 and MyoD expression or resistance to injury susceptibility assessed with laser-induced membrane damage. **(Middle)** Scaffold engineered muscles are created by embedding muscle progenitor cells at high density in a hydrogel. Cells remodel the hydrogel to generate 3D tissues packed with aligned functional myotubes. **(Bottom)** Scaffold-free engineered muscles created by seeding a monolayer of cells on laminin coated plates. The cells secrete sufficient ECM and progressive lift off the plate or “delaminate” and roll up to generate small 3D tissues. Both scaffold and scaffold-free tissues can be utilized for multiple assays with multiple functional output for up to 4 weeks.

To overcome the limitations of 2D cell culture, several 3D skeletal muscle culture models have been developed over the past 30 years (Khodabukus et al., 2018; Wang J. et al., 2019). Two culture methods have been predominantly utilized: (1) hydrogel and (2) self-organized/assembled tissues (Figure 1). Both methods aim to create a biomimetic muscle microenvironment that provides cells with the appropriate extracellular matrix (ECM) and the biological and mechanical signals to promote rapid muscle development, maturation, and function.

### Hydrogels

The majority of engineered tissues are generated from three-dimensional hydrogels derived from natural ECM proteins such as collagen and fibrin. Hydrogels should: (1) provide a high surface area for cell adhesion, (2) provide mechanical support and/or topical guidance to maximize, (3) minimize diffusion distances, and (4) fully degrade once sufficient cell-derived ECM is deposited to generate densely packed muscle tissue supported by its own ECM. Hydrogel based tissues are typically formed from expanded MPCs that are embedded at high density within or on top of these hydrogels. The hydrogels are cast between two fixed anchor points that enable cellular forces to remodel the hydrogel and maintain the tissues under tension that promotes tissue alignment, rapid fusion, and muscle hypertrophy (Vandenburgh et al., 1988; Khodabukus et al., 2018; Wang J. et al., 2019).

### Collagen

The first 3D engineered tissues utilized Type I collagen hydrogels (Vandenburgh et al., 1988; Okano and Matsuda, 1997, 1998; Shansky et al., 1997; Powell et al., 2002), due to Type I collagen being the most abundant ECM protein in skeletal muscle (Kjaer, 2004). Whilst these studies initially utilized the C2C12 cell line, this method has successfully generated tissues from primary rodent (Vandenburgh et al., 2008; Lee and Vandenburgh, 2013) and human (Powell et al., 2002; Brady et al., 2008; Gholobova et al., 2015) cells. While collagen I is the most abundant ECM in skeletal muscle, excessive collagen levels are associated with poor regeneration and function of native muscle (Stearns-Reider et al., 2017). In native muscle, myofibers directly interact with the basal lamina which is rich in collagen IV and laminin but not type I collagen (Thorsteinsdottir et al., 2011). To better model native muscle structure, collagen hydrogels can be combined with Matrigel^TM^, a commercially available basal lamina extract isolated from murine Engelbreth-Holm-Swarm tumors, at the time of tissue formation. Collagen-matrigel hydrogels improve muscle structure but still generate lower contractile forces than fibrin based tissues and consequently are being used less frequently for studies measuring contractile function (Hinds et al., 2011; Sato et al., 2011).

### Fibrin

Fibrin is the major structural component of blood clots that functions to first prevent bleeding and then be completely remodeled, resorbed, and replaced over time making it an ideal substrate for tissue engineering (Ahmed et al., 2008). The main disadvantage of fibrin is the significant lot-to-lot variability in tissue function and gel degradation rate which can be overcome by lot testing and regulation of fibrinolysis with cross-linkers and anti-fibrinolytics, respectively (Khodabukus and Baar, 2009). Tissues engineered from fibrin alone have specific contractile force generation significantly higher than collagen-matrigel tissues (Huang et al., 2005, 2006) and can be further enhanced by the addition of matrigel to generate engineered muscle tissues with the highest reported contractile function (Hinds et al., 2011; Juhas et al., 2014; Madden et al., 2015; Khodabukus et al., 2019). The increase in force generation is due in part to fibrin being several orders of magnitude less stiff than collagen (Collet et al., 2005; Yang et al., 2007) and fibrin gels having muscle-like stiffness (Chiron et al., 2012). Substrate stiffness is a key regulator of tissue dependent gene transcription programs (Engler et al., 2006), with muscle-like stiffness increasing myogenic gene expression and promoting muscle maturation (Engler et al., 2004).

### Scaffold-Free/Self-Assembled Muscle Tissues

An alternative approach to the use of hydrogels/scaffolds is to allow cells to secrete their own ECM and self-organize into a 3D tissue. The first self-organized engineered muscle used saran wrap substratum upon which MPCs were seeded with fibroblasts to ensure sufficient ECM deposition to enable tissue self-assembly (Strohman et al., 1990; Li et al., 2011). This model was then improved by seeding cells onto a PDMS substratum coated with laminin to support cell adhesion and reduce the number of fibroblasts required to generate sufficient ECM to support tissue formation (Dennis and Kosnik, 2000; Dennis et al., 2001; Kosnik et al., 2001; Larkin et al., 2006a, b). Further improvements to this model by utilizing aligned micropatterned surfaces to both quicken myoblast fusion and myotube alignment resulted in greater muscle differentiation (Lam et al., 2009). More recently, self-organized muscle cell sheets have been generated using the thermoresponsive polymer poly(N-isopropylacrylamide) (Takahashi and Okano, 2015; Takahashi et al., 2018). The cell sheets can be detached from culture plates by lowering temperature and used to engineer multi-layered tissue sheets comprised of muscle, vascular, or neuronal cells (Nagamori et al., 2013; Ngo et al., 2013; Takahashi et al., 2013). The benefits of these self-organized models are the tissues being encased entirely in cell secreted ECM and circumventing the lot variability of commercially available ECM proteins. However, the longer time to tissue formation and the inability of this model to easily be automated have resulted in hydrogel methods being more frequently used. A further limitation of self-assembled tissues is the small tissue size which hinders translational studies, though the ability to stack cell sheets or engraft multiple tissues together may overcome this issue.

### Tissue-Engineered Muscle Function

Tissue engineered muscles replicate basic muscle contractile physiology such as force-length relationships and positive force-frequency relationships (Dennis and Kosnik, 2000; Huang et al., 2005; Hinds et al., 2011). However, their twitch:tetanus ratio, contractile kinetics (i.e., time to peak tension and half-relaxation time), and specific force generation are more consistent with embryonic and neonatal skeletal muscle values (Close, 1972). The developmentally immature contractile properties reflect the developmentally immature transcriptome and protein isoform expression seen in engineered tissues. While myotubes with engineered tissues have more mature gene expression and greater hypertrophy than myotubes cultured in 2D and undergo progressive hypertrophy in culture (Rao et al., 2018), even after 4 weeks of culture myotubes resemble developmentally immature or long-term denervated fibers (Kern et al., 2004). As discussed below, the incorporation of additional cell types and electro-mechanical stimulation can further increase muscle hypertrophy and contractile function. However, significant advances are required to generate adult-like engineered muscles within the shortened timeframe that would be most attractive to researchers.

## Skeletal Muscle Models for Studying Muscle Growth and Plasticity

Skeletal muscle has a remarkable level of plasticity that enables muscle to adapt to several physiological stressors such as changes in contractile activity, mechanical load, nutritional state, and hypoxia. Studying these processes in isolation *in vivo* is extremely difficult and confounding factors can hinder interpretation of the results. As discussed below, tissue-engineered skeletal muscle models have the potential to study many of these factors in isolation to not only better understand these mechanistic processes but to further enhance the maturation of engineered muscle tissues.

### Skeletal Muscle Fiber-Types

**FIGURE 2:**
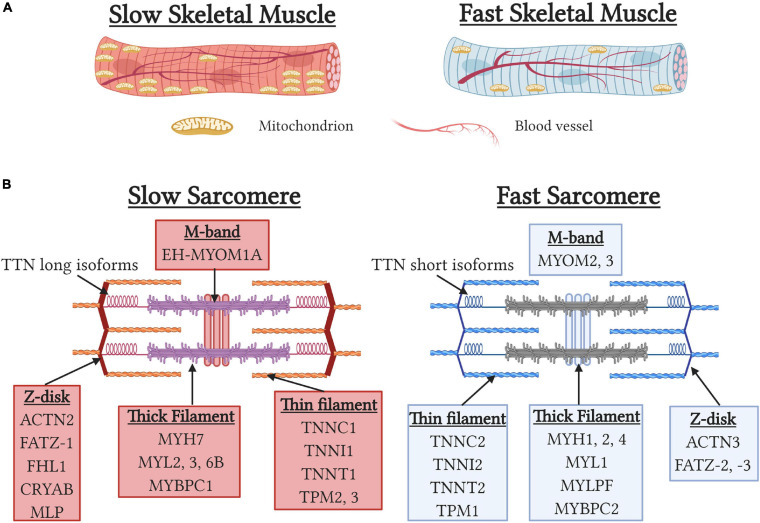
Structural differences between slow and fast-fiber type muscles. **(A)** Schematic depicting gross morphological differences between slow and fast skeletal muscles. Note the higher levels of mitochondria and capillary density associated with slow skeletal muscles. **(B)** Sarcomeric gene specialization and enrichment in slow and fast myofibers.

Skeletal muscle can be classified as type 1 slow-twitch (ST) or type 2 fast-twitch (FT) fiber types based on myosin heavy chain (MYH) isoform expression. While ST fibers express type I myosin (MYH7), FT fibers can be further classified into three subtypes, IIa (MYH1), IIx (MYH2), and IIb (MYH4), resulting in four fiber-type classifications. However, Type IIb fibers which possess the fastest contractile phenotype, are absent in human skeletal muscle resulting in humans only possessing three fiber-types (Schiaffino, 2010). During development and muscle regeneration, the embryonic (MYH3) and neonatal (MYH8) MYH isoforms are sequentially expressed before being down regulated and replaced by the adult MYH isoforms (Chargé and Rudnicki, 2004). Importantly, approximately 25% of muscle fibers are “hybrid” and express two or more MYH isoforms that arise to provide a functional continuum for optimal contractile performance or reflect transitionary states during development and regeneration (Medler, 2019). In addition to MYH isoform, slow and fast isoforms of multiple sarcomeric and calcium-handling proteins are found in a graded fashion in fiber-types to regulate speed of contraction. Functionally, slow contractile and calcium-handling isoforms result in slower contractile kinetics and importantly permit more energy efficient contraction by utilizing less ATP to generate equivalent contractile forces than fast muscle fibers (Bottinelli et al., 1994; Stienen et al., 1996). Additionally, ST fibers possess sarcomeric isoforms of titin, nebulin, and myomesin that contribute to the increased width of the z-disk and help to stabilize muscles during muscle contraction (Yamaguchi et al., 1985; Agarkova et al., 2004; Prado et al., 2005) (Figure 2). Functionally, these adaptations result in ST fibers being less prone to contraction-induced muscle damage (Choi and Widrick, 2010) and contribute to the increased disease severity of FT fibers seen in Duchenne muscle dystrophy (Webster et al., 1988). Lastly, type I and IIa fibers are characterized by high mitochondrial content, increased myoglobin levels, and high capillary density to maximize oxygen delivery to support more oxidative metabolism. Importantly, mitochondrial specialization occurs between fiber-types with mitochondria in ST fibers adapted to maximize fatty acid oxidization and reduced mitochondrial transition pore opening to prevent cell death due to the chronically elevated calcium levels in ST fibers (Picard et al., 2012). In contrast, mitochondria in FT fibers have a 10-fold greater ability to oxidize of glycerol-3-phosphate to help maintain a balanced redox state (Picard et al., 2012). ST fibers also have increased reactive oxygen species scavenging capacity due in part to increased antioxidant enzyme levels (Loureiro et al., 2016) and increased NADPH generation due to increased isocitrate dehydrogenase two expression and consequent regulation of the tricarboxylic acid cycle (Murgia et al., 2015).

Over the past 30 years, significant advances in elucidating the complex molecular and transcriptomic regulation of fiber-type have been achieved. A combination of epigenetic imprinting, neuronal activity, oxygen tension, environmental factors, and metabolic and hormonal influences regulate signaling pathway cascades and transcription factor activity (Hoppeler, 2016). A key regulator of fiber-type specification is intracellular calcium concentrations, with slow tonic motor neuron activity promoting sustained elevated calcium levels that activate calcineurin and calmodulin kinase (Meissner et al., 2001; Kubis et al., 2003). This activation then results in increased activity of NFAT (Swoap et al., 2000; Kubis et al., 2002, 2003; Mccullagh et al., 2004; Calabria et al., 2009; Ehlers et al., 2014) and MEF2 (Wu et al., 2000), and decreased class II HDAC activity (Potthoff et al., 2007) to promote slow sarcomeric and oxidative metabolism gene expression. The four isoforms of NFAT contribute to muscle fiber-type with NFATc1-4 expressed in ST fibers, NFATc2-4 expressed in type IIa fibers, and IIb fibers only expressing NFATc4 (Calabria et al., 2009). In addition to regulation by calcineurin, the transcription factor Prox1 increases expression of NFATc1-3 (Kivela et al., 2016), represses multiple members of the fast transcription program (Petchey et al., 2014), and is required for typical slow fiber distribution. Elevated calcium levels also promote mitochondrial biogenesis and oxidative phosphorylation via activation of the transcription factors PGC1α, PGC1β, PPARβ, and PPARδ (Jornayvaz and Shulman, 2010; Phua et al., 2018). Overexpression of PGC1α (Handschin et al., 2007; Rasbach et al., 2010; Summermatter et al., 2012), PPARβ (Schuler et al., 2006), and PPARδ (Wang et al., 2004) result in fast-slow fiber type and alter sarcomeric and calcium-handling gene expression demonstrating the interrelationship between metabolism, calcium-handling, and sarcomeric transcriptome. Additionally, overexpression of TEAD1, a member of the Hippo pathway, induces fast-to-slow fiber type conversions (Tsika et al., 2008). Conversely, a slow-to-fast transition occurs in the presence of thyroid hormone (Zhang et al., 2014) or deletion of VGLL2 (Honda et al., 2017) by disruption of TEAD1. Whilst the molecular transduction pathways leading to fast muscle fiber-types are less well characterized, genetic regulation of fast muscle fiber-type is well established. Fast muscle fiber neural input is linked to increased HIF1α content (Lunde et al., 2011), which is known to increase glycolytic gene expression, and decreased MyoD phosphorylation (Ekmark et al., 2007). MyoD is enriched in fast muscle fibers (Hughes et al., 1997) and repressed by the key slow-fiber type regulator NFATc1 (Ehlers et al., 2014) but its deletion only results in mild fiber-type switching suggesting that, whilst important, MyoD is not a master regulator of fast-fiber transcription. The master regulators of the fast muscle program are the Six1, Six4, Eya1 transcription factor complex which promote fast muscle gene expression (Grifone et al., 2004; Niro et al., 2010) and the transcriptional repressor Sox6 which inhibit slow muscle gene expression and increases fast muscle gene expression (Hagiwara et al., 2005, 2007; An et al., 2011; Quiat et al., 2011; Wang et al., 2011; Sakakibara et al., 2014). In humans, increases in fast muscle fiber-types are seen in donors with polymorphisms in HIF1α that increase HIF1α transcriptional activity (Ahmetov et al., 2008). Similarly, polymorphisms that decrease levels of the fast-fiber specific z-disk protein α-actinin3 are associated with increased calcineurin activity and endurance performance indicating that regulators of muscle fiber type can regulate human muscle performance (Yang et al., 2003; Eynon et al., 2012; Seto et al., 2013).

### Role of Neural Input on Muscle Fiber Type *in vivo*

Complete muscle development and maintenance of adult muscle mass and fiber-type requires functional innervation. A key difference between slow and fast fiber-types is the motor input received with the frequency neural impulses differing between slow (10–30 Hz) and fast (50–100 Hz) muscle fibers (Hennig and Lomo, 1985; Eken et al., 2008). Additionally, slow fibers are active for greater periods of time and receive a far higher number of neural impulses per day than fast muscle fibers. The predominant role of neural input in regulating muscle fiber-type was first described in 1960 (Buller et al., 1960) and detailed in multiple elegant studies in the 1960s and 1970s (Close, 1969; Barany and Close, 1971; Hoh, 1975). Specifically, the contractile properties of slow muscles become faster when reinnervated by a fast nerve and fast muscles become slower when reinnervated by a slow nerve. The second series of studies used implanted electrodes to determine the role of stimulation frequency, work:rest ratio, and contractile impulse numbers in directing contractile kinetics and fiber-type shifts (Salmons and Sreter, 1976; Eberstein and Pachter, 1986; Eken and Gundersen, 1988; Gorza et al., 1988; Westgaard and Lomo, 1988; Windisch et al., 1998). The seminal finding of these studies is that neural input firing pattern is the predominant neural factor regulating muscle fiber-type and not secreted neurotrophic factors (Salmons and Sreter, 1976). Overall, these studies showed that increasing stimulation frequency, decreasing the work:rest ratio, and decreasing the total number of delivered impulses resulted in shifts to a faster-fiber type. Critically, both experimental models revealed that each individual muscle has an “adaptive range”, the degree of plasticity is species dependent, and long time periods (>3 months) are required for more definitive fiber-type changes. This adaptative range can be expanded by thyroid levels, with hypothyroidism and hyperthyroidism promoting greater slow and fast fiber-type shifts respectively (Kirschbaum et al., 1990; Caiozzo et al., 1998, 2000; Di Maso et al., 2000; Zhou et al., 2019). Overall, the typical adaptive ranges of mouse ST fibers permit expression of type I, IIa, and IIx but not IIB fibers, while mouse FT fibers can express Type IIa, IIx, and IIB fibers but not type I fibers.

### Role of Neural Input on Muscle Fiber Type *in vitro*

The role of distinct neural inputs can be studied in culture by field electrical stimulation without potential confounding effects such as regenerative ability, compensatory hypertrophy, and animal locomotion. In 2D cultures, these studies have demonstrated that slow or fast-like stimulation patterns can increase isoform specific sarcomeric and calcium-handling proteins, and induce muscle hypertrophy (Wehrle et al., 1994; Hamalainen and Pette, 1997). The number of impulses and period of contractile activity must be regulated to prevent detachment of contracting myotubes in 2D culture. In contrast, in engineered muscle tissues the 3D environment supports tissue contraction and permits long-term electrical stimulation. However, long-term field electrical stimulation can induce electrochemical damage resulting in tissue damage and ultimately cell death. Electrochemical damage can be minimized by utilizing bipolar impulses and optimizing impulse parameters (i.e., electric field and pulse width) based on tissue excitability to enable adult-like long-term, chronic electrical stimulation without electrochemical damage for at least 2 weeks (Donnelly et al., 2010; Khodabukus and Baar, 2012, 2015c). Clinically, rheobase and chronaxie have been utilized to prevent tissue damaged in chronically denervated patients undergoing neuromuscular electrical stimulation (NMES) therapy (Pieber et al., 2015). The excitability of engineered tissues are similar to that of long-term denervated muscle and thus have potential to be used as an *in vitro* model to study factors regulating excitability and to screen factors that promote muscle excitability (Dennis and Dow, 2007; Khodabukus and Baar, 2012). In patients, NMES results in increased muscle cross-sectional area and tissue functionality in long-term denervated tissue (Boncompagni et al., 2007; Kern et al., 2010; Kern and Carraro, 2014; Carraro et al., 2015). In engineered muscle tissues, electrical stimulation increases force generation, muscle hypertrophy, and MHC and dystrophin protein levels in engineered tissues (Huang et al., 2006; Khodabukus and Baar, 2012, 2015c; Ito et al., 2014; Khodabukus et al., 2015, 2019). Like native muscle (Baar and Esser, 1999; Terzis et al., 2008; Drummond et al., 2009), these increases in muscle hypertrophy are associated with increased mTORC1 activity and can be inhibited by the mTOR inhibitor rapamycin. These studies also show that electrical stimulation of engineered muscle tissues for 1 or 2 weeks does not induce transformative changes in muscle size and that additional factors and/or time are required to attain adult muscle size.

To date, relatively few studies have determined the fiber-type impacts of different electrical stimulation protocols on engineered tissue function. The first study showed differential functional responses between muscles engineered from cells isolated from fast and slow muscles when electrically stimulated with biomimetic fiber-type neural input protocols (Huang et al., 2006). Specifically, engineered slow tissues increased force generation but did not alter their contractile kinetics in response to slow-fiber mimetic electrical stimulation. In contrast, slow-fiber mimetic stimulation in TA tissues did not increase force generation but did slow contractile kinetics. The fast fiber-type protocol did not change any parameters in either slow or fast engineered tissues, potentially due to the number of contractions being insufficient to induce functional changes or the lack of factors such as T3 which are critical for the fast-fiber type program. Human engineered tissues increase force generation and hypertrophy when subjected to a fast-like periodic intermittent electrical stimulation protocol with slow-like 1 or 10 Hz frequencies (Khodabukus et al., 2019). The 10 Hz but not the non-physiological 1 Hz protocol induced a quickening of half-relaxation time which was associated with increased fast CSQ and SERCA gene expression indicating that initial fiber-type shifts can be studied in human tissues. To date, the greatest fiber-type shift has been shown in C2C12 tissues where the role of contraction duration in inducing a slow fiber-type transition was defined (Khodabukus et al., 2015). When keeping the stimulation frequency, work:rest ratio, and total number of impulses received per day consistent, contraction lengths of greater than 6 s were required to induce more complete fast-to-slow MYH shifts. Fast-to-slow isoform switching of each troponin isoform, CSQ, and SERCA were independent of contraction length changes, suggesting differential regulation of MYH and other contractile proteins. These changes in protein abundance correlated to functional changes, with the slow electrical stimulation paradigm inducing slowing of contractile kinetics and increased fatigue resistance. Overall, these studies demonstrate the ability of engineered muscle tissues to study the fiber-type changes induced by electrical stimulation. However, while generation of slow fiber-types has been demonstrated, further optimization of the culture conditions and electrical stimulation protocols are required to generate fast fiber-type tissues.

### Motor Innervation of Engineered Muscle Tissues

**FIGURE 3:**
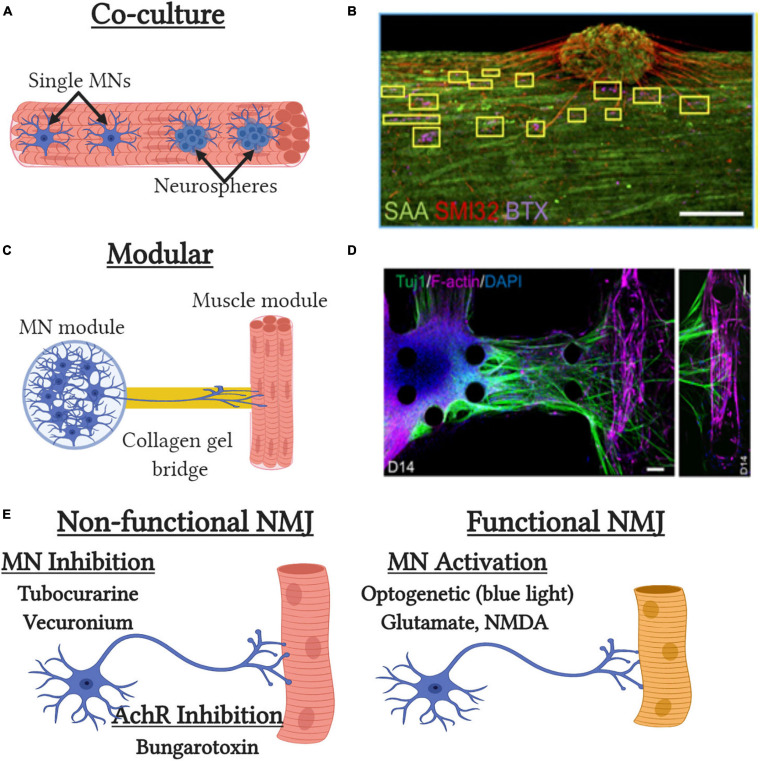
Tissue-engineered neuromuscular junction (NMJ) models. **(A)** Traditional co-culture NMJ models where single MNs or neurospheres are embedded with muscle cells at the time of tissue formation. **(B)** Representative image of the co-culture NMJ model (Afshar Bakooshli et al., 2019) with immunofluorescent staining of neurite extensions (SMI32), acetylcholine receptors [α-bungarotoxin (BTX)] and muscle sarcomeres [sarcomeric α-actinin (SAA)]. Scale bar, 200μm. **(C)** Modular NMJ muscle model where motor neurons (MNs) and engineered muscle tissues are cultured in separate compartments and connected by a collagen gel which supports neurite extension. **(D)** Representative image of the modular NMJ model (Osaki et al., 2018b) with immunofluorescent staining of neurite extensions (TUJ1) and myotubes [filamentous(f)-actin]. Scale bars, 100 μm. **(E)** Schematic depicting pharmacological and genetic methods to assess neuromuscular junction (NMJ) functionality *in vitro*.

While field electrical stimulation can mimic neural input, it does not faithfully recapitulate EC-coupling or model the complex physical and chemical interactions between muscle and nerves. Motor innervation is essential for complete muscle development and maintenance of muscle mass and defects in the neuromuscular junction (NMJ) result in multiple neuromuscular diseases such as myasthenia gravis (MG) and amyotrophic lateral sclerosis (ALS) (Rudolf et al., 2016; Cappello and Francolini, 2017). The first reports of NMJ formation were reported over 40 years in co-cultures of Xenopus myoblasts and embryonic neurons (Anderson and Cohen, 1977; Anderson et al., 1977). Similar studies utilizing rodent cells show markers of muscle maturation such as fetal to neonatal MHC isoform transitions, increased sarcomere structural maturation, and acetylcholine receptor (AchR) clustering (Das et al., 2007, 2010; Guo et al., 2010). However, in these studies AchR clustering does not co-localize with nerve terminals, as seen in embryonic development (Witzemann, 2006), the NMJs fail to replicate mature pretzel-like morphology, and no definitive evidence of electrical transmission provided suggesting incomplete NMJ development and maturation. In the past decade, substantial progress has been made in generating motor neuron-like progenitors from hiPSCs to permit studies utilizing human cells (Patani et al., 2011; Amoroso et al., 2013; Faravelli et al., 2014; Sances et al., 2016). These cells when co-cultured with primary or hiPSC-derived myotubes demonstrate AchR clustering but more importantly show functional NMJ formation (Demestre et al., 2015; Puttonen et al., 2015; Toli et al., 2015). Specifically, motor neuron-induced muscle contraction can be induced by specific motor neuron glutamatergic excitation by glutamate or N-Methyl-D-aspartate or inhibited by tubocurarine, an anti-cholinergic drug that prevents neural transmission (Figure 3E). Alternatively, muscle contraction can be induced optically by transduction of motor neurons with the light-sensitive ion channel channelrhodopsin2 (ChR2) (Steinbeck et al., 2016). Expression of ChR2 permits specific optical activation of motor neurons with blue light and subsequent myotube contraction if functional NMJs are present. Importantly, the use of hiPSCs allows the modeling of multiple human neuromuscular disease such as MG, spinal muscular atrophy (SMA), and ALS which would not be possible due to the post-mitotic state of adult motor neurons (Dimos et al., 2008; Ebert et al., 2009; Sances et al., 2016).

The first 3D tissue engineered muscle motor-neuron co-cultures utilized neonatal rat MPCs and embryonic spinal cord explants (Larkin et al., 2006b). Neural projections from the explants extended into the engineered muscle and preserved their structure to enable neural specific stimulation of the resulting tissue. Spinal explants increased force generation, ∼25% of which could be achieved by direct neural stimulation, indicating incomplete innervation of all myotubes and/or the presence of non-functional NMJs. Mixing of rat motor neurons with rat MPCs at time of tissue formation also results in improved contractile force and muscle structure, though the level of functional NMJ generation was not tested (Martin et al., 2015). Incorporation of hiPSC-derived neurospheres, that contain motor neurons and other supporting cell types, into primary human engineered muscle tissues results in successful generation of innervated myotubes as assessed by glutamate stimulation (Afshar Bakooshli et al., 2019) (Figures 3A,B). Neurosphere co-culture also resulted in shifts to more developmentally mature AchR subunit expression and more adult-like AchR structure then that seen in 2D cultures. While motor neuron-muscle co-cultures clearly increase functionality and muscle structure, it is still unclear what effects are due to increased contractile activity and secreted neurotrophic factors. The synaptogenic factor agrin can induce AchR formation, AchR clustering, and increase force generation almost 2-fold (Bian and Bursac, 2012). Similarly, the neurotrophic factor neuregulin-1 induced AchR subunit isoform maturation though its role in regulating muscle function unknown (Afshar Bakooshli et al., 2019). The most recent models to study NMJ formation in 3D tissues utilize neural cells and engineered muscle tissues in two distinct modular compartments (Figures 3C,D). These compartments are then attached by a collagen gel bridge that supports and guides neural cell migration and neurite extension into the engineered muscle tissue. Importantly, specific activation of the neural compartment can induced optogenetically by overexpression of ChR2 and engineered muscle contractile function assessed by pillar deformation (Uzel et al., 2016; Osaki et al., 2018b; Vila et al., 2019). Thus in the presence of functional NMJ formation, optical stimulation of the neural compartment induces contraction of the engineered muscle compartment. These models have been used to successfully model impaired NMJ function in ALS and MG (Osaki et al., 2018b; Afshar Bakooshli et al., 2019; Vila et al., 2019) and to identify and validate prospective clinical drug candidates such as rapamycin and bosutinib for ALS (Osaki et al., 2018b). Overall, significant progress has been made with innervation of engineered muscle tissues over the past 5 years and they possess great potential as more biomimetic models for muscle disease and development. However, significant improvements in NMJ maturation are still required as well as more detailed assessment of NMJ electrophysiology, structure, and function.

### Effect of Cell Source on Fiber-Type

A fundamental question underlying muscle plasticity is if the satellite cells that make typically slow (e.g., SOL) or fast (e.g., tibialis anterior) muscle fibers are distinct and predisposed to generate a specific fiber-type. As discussed above, the adaptive ranges of muscle fibers suggest that fiber-type is predetermined. Slow fiber-type muscles contain a higher level of satellite cells than fast fiber type tissues (Gibson and Schultz, 1982; Putman et al., 2001; Mackey et al., 2009). This difference is further exacerbated with aging due to a greater loss of SCs in fast muscle fibers (Verdijk et al., 2007, 2014), which correlates to the greater loss of function seen in fast muscles in sarcopenia (Deschenes et al., 2013; Purves-Smith et al., 2014). Satellite cells isolated from slow-fiber type muscles have increased transplantation efficiency and thus are likely more translationally relevant for future SC transplantation therapies (Collins et al., 2005). The existence of intrinsic differences between satellite cells from fast and slow muscles is further suggested by the finding that electrical stimulation of regenerating slow SOL and fast EDL muscles with the same slow stimulus pattern in the absence of innervation leads to widespread slow MYH expression in regenerated SOL but only limited expression of slow MYH in regenerated EDL (Kalhovde et al., 2005). Additionally, the altered MYH isoform profile of single fibers isolated from long-term denervated slow and fast-fiber type muscles remain distinct and do not converge to similar MYH isoform expression (Patterson et al., 2006). Traditional 2D cell cultures of cells isolated from slow and fast muscles show preferential expression of fiber-type MYH expression and displayed expected adaptive ranges to fiber-like electrical stimulation (Wehrle et al., 1994; Rosenblatt et al., 1996; DiMario and Stockdale, 1997). In 3D culture, muscles engineered from MPCs isolated from rats (Huang et al., 2006) or mice (Khodabukus and Baar, 2015a) retain the contractile and metabolic properties of the muscles from which they were derived. Specifically, expected isoform shifts in multiple sarcomeric, calcium-handling, and metabolic proteins are seen. However, due to the lack of neural input these shifts are less distinct than seen in innervated adult muscle fibers and more closely resemble long-term denervated myofibers. Interestingly, these changes are linked to changes in transcriptional master regulators such as Sox6 which is more abundant in myotubes derived from fast muscles (Khodabukus and Baar, 2015a). Overall, these studies demonstrate that the satellite cells generating fast and slow muscles are distinct and result in long-term differences in metabolism and function both *in vitro* and *in vivo*.

### Role of Mechanical Load on Engineered Muscle Size and Function

Skeletal muscle function and size is dependent upon the load placed upon it as evidenced by loss of muscle mass and strength following immobilization, bed rest, spaceflight (Haddad et al., 2006; Baldwin et al., 2013), and gain of muscle mass and function with resistance exercise (Hughes et al., 2018; Jorgenson et al., 2020). Mechanical load is modeled *in vitro* by the application of stretch to tissue cultures using deformable membranes (Sim et al., 2007; Pavesi et al., 2015) or custom-made bioreactors (Dennis et al., 2009; Rangarajan et al., 2014) to induce programmable levels of stretch in custom-made regimes. Most studies utilize custom-made systems and unique stimulation protocols that hinder comparisons between studies. To date, *in vitro* mechanical stimulations of engineered muscle have aimed to replicate: (1) the continual increase in muscle length seen during development (ramp stretch/stimulation); and; (2) muscle strain that occurs during locomotion and exercise (cyclic stretch/stimulation). Ramp stretch results in concomitant proliferation and differentiation of MPCs (Vandenburgh and Karlisch, 1989) which may be due in part to the secretion of the IGF-1 splice variant mechanogrowth factor (Cheema et al., 2005). Application of ramp stretch for only 45 h induces significant muscle hypertrophy and increases in contractile force generation in C2C12 engineered tissues (Aguilar-Agon et al., 2019). Applying both ramp and cyclic stretch induced significant myotube hypertrophy in engineered human tissues (Powell et al., 2002) and increased glucose uptake in avian tissues (Hatfaludy et al., 1989). The seminal *in vitro* work of Vandenburgh showed that cyclical stretch induces muscle hypertrophy and increases protein and DNA content (Vandenburgh and Kaufman, 1979, 1981; Vandenburgh, 1982, 1988; Vandenburgh et al., 1989, 1991). Like native muscle following resistance exercise or altered mechanical load (Baar and Esser, 1999; Terzis et al., 2008; Marabita et al., 2016), mechanical stimulation induced hypertrophy of *in vitro* cultures is associated with mTORC1 activation (Baar et al., 2000; Khodabukus et al., 2007; Aguilar-Agon et al., 2019). Conversely, muscle atrophy can be modeled by decreasing engineered tissue length to induce decreases in myotube size and loss of contractile force (Lee and Vandenburgh, 2013). Together, these studies demonstrate that mechanical loading/unloading can model aspects of muscle hypertrophy/atrophy and be used a model system to study these processes. Similar to electrical stimulation studies, no transformative breakthroughs in generating engineered tissues with native-like muscle size or strength have been reported with use of mechanical stimulation alone or in combination with electrical stimulation (Liao et al., 2008).

### Metabolic and Hormonal Control of Muscle Fiber-Type *in vitro*

A key difference between fiber-types is the greater reliance of fast-fiber type muscles on glycolysis and slow-fiber-type muscle on oxidative phosphorylation to meet energy needs. Muscles engineered from fast twitch muscle fibers have increased levels of glycolytic enzymes and fatigue at a greater rate than tissues generated from slow twitch muscle fibers (Khodabukus and Baar, 2015a). Engineered C2C12 tissues cultured with supraphysiological glucose levels promotes a fast fiber-type contractile and metabolic phenotype, permits detectable levels of the fast-fiber specific protein parvalbumin, and increased levels of glycolytic proteins (Khodabukus and Baar, 2015b). Whilst promising, accurate modeling and studying of oxidative metabolism *in vitro* is hindered by the Crabtree effect where *in vitro* cultured cells typically utilize glycolysis to generate ATP despite the presence of abundant oxygen and functional mitochondria (Crabtree, 1929; Marroquin et al., 2007). The Crabtree effect can be circumvented by replacing glucose in the cell culture media with galactose which forces cells to rely on oxidative phosphorylation to meet their energy demands due to galactose requiring 2 molecules of ATP to generate pyruvate and thus producing 0 net ATP from anaerobic glycolysis (Marroquin et al., 2007; Ryall et al., 2015). Galactose culture of primary human myotubes increases mitochondrial enzyme levels, increases basal and maximal oxygen consumption, and increased phosphorylation of AMPK (Aguer et al., 2011) – which when chronically activated results in a fast-to-slow fiber-type transition (Ljubicic et al., 2011). Importantly, these adaptations to galactose culture conditions were not seen in myotubes derived from type II diabetics suggesting that true assessment of metabolic dysfunction in diabetic myotubes may require novel culture conditions (Aguer et al., 2011). In contrast, C2C12 cells do not respond to galactose culture conditions indicating that modeling metabolism in immortalized cell lines should be treated with caution (Elkalaf et al., 2013). Alternatively, supplementing culture media with fatty acids has the potential to promote oxidative metabolism and potentially promote a slow fiber-type phenotype. However, *in vitro* cultures are prone to lipotoxicity due to the low reliance on oxidative phosphorylation and β-oxidation to meet energy demands. In agreement with this, the saturated fatty acid palmitate is known to induce atrophy (Bryner et al., 2012), insulin resistance (Yuzefovych et al., 2010), and cell death (Patkova et al., 2014) in myotubes. Addition of polyunsaturated fatty acids (PUFAs) such as oleic, linoleic, docosahexaenoic, and eicosapentaenoic acid can reduce or prevent these negative cellular effects of palmitate (Bryner et al., 2012; Yuzefovych et al., 2012; Lee et al., 2017; Woodworth-Hobbs et al., 2017). Recently, oleic acid alone was shown to increase MYH7 protein levels, mitochondrial mass, and increase maximal respiration rate in human myotubes supporting the potential role of fatty acids in promoting fiber-type shifts (Watanabe et al., 2020). In contrast, promoting a fast fiber-type *in vitro* has proven to be more difficult due in part to the fact that fast MYH expression occurs in more mature muscles cultures (Cooper et al., 2004). Lactate can increase fast MYH (MYH 1 and 4) gene expression (Tsukamoto et al., 2018) and thyroid hormone upregulates fast SERCA isoform expression (Thelen et al., 1997). Overall, these studies show that fiber-type shifts can be studied *in vitro* by modulation of fuel source and hormone availability but advances in generating fast fiber-type changes are still required.

## Vascularization of Engineered Muscle Tissues

Native skeletal muscle is highly vascularized to provide the oxygen and nutrient supply required to support the high metabolic demands induced by muscle contraction. Hypoxia and impaired cell survival typically occur at a diffusion distance of 150–200μm from blood vessels or in culture media and limits tissue-engineered muscle size (Gholobova et al., 2020a). Satellite cell fate and muscle regeneration is also regulated by vasculature (Abou-Khalil et al., 2009, 2010; Fu et al., 2015), with 50-80% of satellite cells are in near or direct contact with capillaries (Verma et al., 2018). Therefore, vascularized tissue-engineered skeletal muscles are required for accurate modeling of native skeletal muscle *in vitro* and effective cellular therapies *in vivo*.

Vascularization of tissue-engineered skeletal muscles has typically been performed by the incorporation of vascular cells at the time of tissue formation. A key technical limitation with vascularized engineered muscle tissues are the incompatible media requirements of muscle and vascular cells (Gholobova et al., 2015). This has led to compromised media conditions that result in suboptimal differentiation of muscle and/or vascular cells than if cultured in isolation. A second technical limitation of co-culturing muscle and vascular cells is the potential for vascular cells to impede myoblast fusion. This issue can be overcome by 3D bioprinting techniques (Choi et al., 2019), the stacking of muscle-only and vascular-only cell sheets (Nagamori et al., 2013), culturing vascular and muscle cells in distinct compartments (Osaki et al., 2018a), or the coating of vascular cells to differentiated mature muscle tissues (Gholobova et al., 2020b). The formation of stable vasculature requires the inclusion of supporting cell types such as fibroblasts, pericytes, and/or smooth muscle cells (Levenberg et al., 2005; Koffler et al., 2011; Perry et al., 2017). Implantation of pre-vascularized engineered muscle tissues with these supporting cell types accelerates vascular anastomosis, increases blood vessel density, and tissue survival compared to co-cultures alone. While the benefit of forming vascular networks for implantation survival is well established, to date no studies have shown increased muscle function, increased muscle maturation, or enhanced SC quiescence in co-cultured tissues *in vitro*. Additionally, the ability of these vascular networks to enhance nutrient delivery and increase engineered muscle size *in vitro* have yet to be shown.

## Modeling and Treating Muscle Disease

Over the last century, small animal models have been the predominant experimental platform to study disease by genetic manipulations, surgical procedures, or other interventions such as changes made to diet and aging. These studies have given us the majority of the insight we have into the underlying mechanisms of disease and for identifying and pre-clinical validation of novel therapeutics. When assessing all drugs entering clinical trials for all diseases, the successful clinical translation is alarmingly low – with less than 1% of identified drugs making it to the clinic resulting in an estimated cost of $1 billion dollars per drug (Hay et al., 2014). The underlying reasons for this low level of success are multifactorial but include species-specific differences in drug metabolism and toxicity (Dykens and Will, 2007; Shen et al., 2011), animal models not fully recapitulating disease severity in humans (Yucel et al., 2018), animals not accurately modeling human pharmacogenomics (Weinshilboum and Wang, 2017), and epigenetic regulation of disease severity (Lamar and Mcnally, 2014). The ability to generate functional human tissue-engineered organs has potential to address some of the limitations of animal studies and provide complementary methods to improve successful drug clinical translation.

### Duchenne Muscular Dystrophy

Duchenne muscular dystrophy (DMD) is a fatal X-linked recessive disorder that affects approximately 1 in 5000 male births due to mutations in the dystrophin gene. Dystrophin is part of the dystrophin-glycoprotein complex (DGC) that functions to efficiently transmit contractile force to the ECM and stabilize the sarcolemmal membrane during contraction to prevent muscle damage. The increased susceptibility to muscle injury due to compromised membrane stability and impaired regenerative response of satellite cells results in constant cycles of muscle degeneration and regeneration. Ultimately, this leads to progressive muscle weakness, loss of ambulation, and fatal respiratory failure by the age of 20. Currently, there is no curative treatment with standard-of-care corticosteroid (prednisone and deflazacort) therapy extending life expectancy up to the fourth decade by delaying disease progression (Bushby et al., 2010).

The most widespread animal model of DMD, the mdx mouse model, arose due to spontaneous mutation in exon 23 on the dystrophin gene in C57/BL10 mice. Like DMD patients, mdx muscles undergo consistent rounds of degeneration and regeneration, display increased susceptibility to eccentric contractions, and display abnormalities in SC function. However, these mice do not show more severe disease phenotypes such as severe muscle weakness, early mortality, cardiac dysfunction and only display significant fat and fibrotic replacement at old age (20–24 months) (Yucel et al., 2018; Aartsma-Rus and Van Putten, 2019). In the past two decades, additional dystrophin-deficient mice have been generated on the C57/BL6 (e.g., mdx^*cv*2–5^) and on the DBA/2J backgrounds (Yucel et al., 2018; Aartsma-Rus and Van Putten, 2019). The mdx^*cv*2–5^ mice have mutations in a range of exons but still demonstrate the lack of functional weakness seen in traditional mdx model. In contrast, the hDMDdel45D2/mdx mouse displays a stronger dystrophic phenotype and displays contractile weakness potentially improving the efficacy of pharmacological therapeutic validation studies (Coley et al., 2016; Young et al., 2017; Van Putten et al., 2019). Mouse models that more accurately replicate human disease severity have required knockout of both dystrophin and utrophin (Deconinck et al., 1997; Grady et al., 1997). These double knockout mice show severe functional and regenerative defects in skeletal muscle, cardiac, and bone tissues. Alternatively, knocking out the telomerase gene in mdx mice (mdx/mTR) induces a more severe muscle (Sacco et al., 2010) and cardiac (Mourkioti et al., 2013) disease phenotype. Together, these double knockout mice provide more severe disease models with which to study dystrophin deficiency and the efficacy of novel therapeutics. Advances in genome editing have permitted the generation of transgenic mice that model disease mutations in human hotspots and allow the testing of gene therapies *in vivo*. However, these mice do not replicate the significant role that disease modifiers play in disease severity for DMD. Multiple genetic and epigenetic modifiers have been shown to significantly influence disease severity and corticosteroid efficacy (Pegoraro et al., 2011; Flanigan et al., 2013; Lamar and Mcnally, 2014; Bello et al., 2015). For example, latent TGF-β binding protein 4 (LTBP4) and osteopontin, which modulate TGF-β signaling, significantly impact disease progression and efficacy of corticosteroid treatment (Bello et al., 2015), and inhibition of TGF-β signaling reversing disease phenotypes *in vitro* and *in vivo* (Choi et al., 2016). Therefore, high throughput personalized *in vitro* muscle platforms that accurately model pharmacogenomic responses will be required to generate high fidelity and clinically predictive drug screens. The mdx mouse model was used as the preclinical system to validate the current standard of care drugs prednisolone (Hudecki et al., 1993) and deflazacort (Anderson et al., 1996). More recently, these models have been used to validate the antisense oligonucleotide treatments eteplirsen (Mendell et al., 2013) which was approved by the FDA in 2016 (Mendell et al., 2013; Stein, 2016; Khan et al., 2019). The use of more severe mdx models in combination with high fidelity human *in vitro* models may lead to more successful clinical translation and drug discovery efforts.

To date, two 3D *in vitro* skeletal muscle models of DMD have been reported using primary cells. The first utilized immortalized dystrophic mouse myoblasts and identified 11 compounds that increased contractile force generation (Vandenburgh et al., 2008). More recently, engineered muscle sheets derived from human primary DMD myoblasts demonstrated decreased fusion ability, decreased myotube size, and produced less force compared to healthy controls after 6 days in differentiation media (Nesmith et al., 2016). Whilst promising, these studies did not describe more mature disease markers such as evidence of degeneration/regeneration, fibrosis or fat replacement or increased susceptibility to contraction-induced injury that will be critical to study DMD pathology and treatment efficacy *in vitro*. Widespread *in vitro* personalized medicine models of DMD or any other myopathy will require the use of hiPSC-derived muscle due to the ethical considerations and proliferative limitations of taking muscle biopsies from myopathic patients. To date, the majority of hiPSC studies of DMD have been performed in 2D cell culture. These studies have demonstrated disease phenotypes such as calcium overload (Shoji et al., 2015), fusion deficits, increased creatine kinase release (Young et al., 2016), and elevated BMP/TGF-β signaling (Choi et al., 2016). Interestingly, fusion deficits are not always seen in hiPSC cultures and appear to be dependent upon media conditions or cell surface marker selection that potentially prevent fusion deficits (Hicks et al., 2018). Recently, a high throughput drug screen in 2D iPSC-derived myotubes identified ginsenoside and fenofibrate as factors that improved hiPSC myoblast fusion *in vitro* and improved mdx morphology and function *in vitro* (Sun et al., 2020). In addition to pharmaceutical screening, engineered tissues can be used to optimize guide RNA design for highly efficient and maximal functional return for CRISPR-Cas9 mediated exon skipping in engineered DMD hiPSC-derived cardiac tissues (Long et al., 2018). A further advantage of hiPSC cell models is the ability to generate increasingly complex disease models by the addition of multiple cell types. In particular, the NMJ pathophysiology in DMD is poorly studied and has the potential to be studied in hiPSC-derived tissues. The generation of multi-cellular 3D muscle tissues holds promise for studying complex tissue interactions in healthy and diseased states (Maffioletti et al., 2018; Mazaleyrat et al., 2020). Overall, the iPSC-derived disease models have significant potential for generating personalized medicine platforms, disease specific drug screening, and studying pathogenic cellular or organ-organ crosstalk in a modular fashion.

### Volumetric Muscle Loss

Skeletal muscle regenerative capacity can be overwhelmed by extensive muscle loss seen with trauma, blast injuries, and surgical resection. In animal models, this is typically modeled by the surgical removal of 20-40% of muscle mass (Sicari et al., 2014; Corona et al., 2017; Quarta et al., 2017). This level of muscle loss results in irrecoverable loss of muscle function and mass with fibrotic replacement of the lost muscle tissue (Corona et al., 2016). In contrast, other injury models such as snake venom, ischemia-reperfusion, crush, and repeated eccentric contractions in healthy skeletal muscle typically result in return of function 1–2 months post injury. The incomplete muscle regeneration in VML injuries is likely due to the ablation of muscle fibers and their associated SCs, the loss of ECM and associated biochemical and mechanical guidance cues, and the extensive and prolonged inflammatory response to VML injury (Greising et al., 2017; Aguilar et al., 2018; Corona et al., 2018; Larouche et al., 2018). The only current clinical option for VML, autologous tissue transfer, is ineffective due to high donor site morbidity and graft failure. Recent clinical trials utilizing decellularized ECM, which promote vascularization and an anti-inflammatory healing response, have shown limited therapeutic efficacy due to the lack of significant *de novo* muscle formation necessitating novel therapeutic approaches (Sicari et al., 2014).

Injection of satellite cells alone into VML injury models does not support muscle regeneration, highlighting the need for a satellite cell niche and myofiber guidance for successful regenerative outcomes (Quarta et al., 2017). In non-VML injury models, successful transplantation of SCs is increased by delivering SCs within a native-like niche such as native myofiber fragments (Marg et al., 2014) or tissue engineered muscle constructs (Juhas et al., 2014). In VML injury models, injection of satellite cells in combination with muscle resident cells (which include endothelial cells and mesenchymal progenitors) attached to artificially engineered muscle fiber scaffold results in retention of SCs and functional regeneration of the injured muscle (Quarta et al., 2017). Incorporating additional cell types such as endothelial (Levenberg et al., 2005; Koffler et al., 2011; Perry et al., 2017; Choi et al., 2019), neuronal (Das et al., 2020; Kim et al., 2020), and immune (Juhas et al., 2018) cells improves the survival of transplanted 3D engineered tissues due a combination of cellular recruitment and paracrine signaling to promote vascular and neural integration. The key limitation in the clinical use of the engineered tissue replacements is neural integration with the host. Implantation of engineered muscle tissues and incorporation of the host nerve into the implant increases implant contractile function and maturation compared to time-matched *in vitro* controls (Borschel et al., 2006; Dhawan et al., 2007; Williams et al., 2013; VanDusen et al., 2014; Adams et al., 2017). In these studies, AchR clustering and primitive NMJ synaptogenesis can occur in as little as 7 days post-implantation but functional integration with the host system was only seen 3 months post-implantation (Urbanchek et al., 2016). Encouragingly, muscle innervation after VML injury treated with satellite cell-containing construct was significantly enhanced with exercise (Quarta et al., 2017), suggesting a possibility that engineered muscle innervation could be optimized through physical therapy. Importantly, the incorporation of exercise following implantation of these tissues promotes improved muscle recovery and host innervation of the resulting muscle fibers (Quarta et al., 2017) – the key limitation in translating engineered muscle to the clinic. Transplantation of engineered muscle tissues results in their initial degeneration due to the loss of nutrients and hypoxia. Generation of tissues that regenerate *in vitro* not only enables the study of muscle regeneration in controlled conditions but also correlates to tissue survival upon implantation (Juhas et al., 2014, 2018). Tissues generated from adult rat MPCs fail to regenerate *in vitro* but the addition of macrophages supports *in vitro* regeneration and increased regenerative ability and functionality upon implantation *in vivo* (Juhas et al., 2018).

## Discussion

The recent advances and progress made in tissue engineering more biomimetic muscle tissues has provided researchers a novel model to complement traditional 2D cell culture and animal models. Here, we have discussed the utility of these engineered tissues to study muscle physiology, regeneration, exercise, and disease. However, the highest functioning engineered muscle tissues have the functionality of neonatal muscle tissue and do not possess the developmental maturity of adult skeletal muscle (Juhas et al., 2014; Khodabukus et al., 2019). Given that full mouse and human muscle maturation requires 3 months and 18 years respectively, methods to rapidly mature engineered muscle tissues are required. Supraphysiological electrical stimulation of engineered hiPSC cardiac tissues accelerates development to achieve adult-like transcriptomic signatures and mitochondrial levels in 4 weeks (Ronaldson-Bouchard et al., 2018). However, the resulting contractile function was inferior to the highest reported in the field demonstrating incomplete tissue maturation and that paradoxically high tissue function does not always equate to greater maturation. For skeletal muscle, the combined use of electrical and mechanical stimulation, functional innervation, small molecules, and appropriately timed biochemical signals will be required to generate more developmentally mature tissues.

For engineered muscle tissues to be more widely utilized, a shift to serum-free culture conditions is also required to increase reproducibility and permit clinical translation of cellular therapies. This is highlighted by the fact that geographical origin of serum can impact the contractile kinetics and isoform expression of calcium-handling proteins in engineered tissues (Khodabukus and Baar, 2014). Furthermore, batch variations in serum, matrigel, and fibrinogen induce significant functional variations, further adding to reproducibility issues in and between laboratories (Khodabukus and Baar, 2009). The use of serum-free differentiation media utilizing commercially available serum-free supplements such as Ultroser-G (Gawlitta et al., 2007; Fujita et al., 2010) or N-2 (Rao et al., 2018; Khodabukus et al., 2020) have demonstrated the muscle function and structure can be maintained or even improved with serum-free supplements. Further advances will also be required in the generation of more physiologically relevant basal media to provide physiologically relevant levels of saccharides, TCA derivatives, metabolites, and hormones (Cantor et al., 2017). Similarly, the use of synthetic or recombinant extracellular matrices will also be required to improve reproducibility, improve toxicity studies, and for successful clinical translation of cellular or engineered tissue therapies (Nguyen et al., 2017). Lastly, the combinatorial use of small molecules can also be used to enhance muscle function (Selvaraj et al., 2019) but the ability to use these tissues for additional drug screening may be limited due to toxicity issues related to high concentrations organic solvents often used as solvent vehicles.

The majority of studies on engineered muscle tissue function are derived of cultures only containing muscle and contaminating or added fibroblasts. While this enables the ability to isolate muscle-specific effects, native muscle tissue is comprised of multiple cell types that are required for complete muscle development and function. The incorporation of motor neurons (Osaki et al., 2018b; Afshar Bakooshli et al., 2019; Vila et al., 2019), sensory neuron (Colon et al., 2017; Guo et al., 2017), vascular (Levenberg et al., 2005; Perry et al., 2017), immune (Juhas et al., 2018), and other supporting cell types will be required to generate more biomimetic engineered muscles. Furthermore, the ability to generate multiple tissue types and to couple them together will enable researchers to study organ-organ crosstalk in a highly controlled and regulated environment. These multi-organ or human-on-a-chip systems are required for more physiologically relevant drug screens and the identification of unexpected drug toxicity (Maschmeyer et al., 2015; Oleaga et al., 2016; Skardal et al., 2017; Vernetti et al., 2017). For drug screening purposes, more high throughput systems such as 96 well plate platforms using pillar deflection methods to record force generation are required (Vandenburgh et al., 2008; Mills et al., 2019). Additionally, identifying culture conditions that prevent the Crabtree effect and promote the use of oxidative phosphorylation to meet energy demands are required to improve tissue maturation and better assay mitochondrial toxicity – the most common factor for drug toxicity (Dykens and Will, 2007; Marroquin et al., 2007). Lastly, generation of clinically relevant muscle tissues for VML injuries will require the combination of all factors discussed above and novel approaches to promote rapid neuronal and vascular host integration. Specifically, the use of small molecules (Ko et al., 2013; Quarta et al., 2016), innovative engineering and/or surgical techniques (Borschel et al., 2006; Kim et al., 2015; Al-Himdani et al., 2017), and physical rehabilitation therapy (Quarta et al., 2017) in conjunction with novel methods to generate viable human size tissues are essential for VML therapies.

Overall, tissue engineered muscle systems are a powerful tool to study skeletal muscle function, development, plasticity, and disease. These systems can be used for stand-alone studies, add functional translational components, or be used to supplement *in vivo* studies as 2D systems have been for decades. Future advances in tissue maturation, generation of more complex heterocellular tissues, and coupling to other organ systems will generate improved models to study muscle disease and increase the chance of identifying novel biological and pharmaceutical therapeutics.

## Author Contributions

AK wrote, edited, and generated figures for the entire manuscript.

## Conflict of Interest

The author declares that the research was conducted in the absence of any commercial or financial relationships that could be construed as a potential conflict of interest.
